# Biodegradation of Aflatoxin B1 in Maize Grains and Suppression of Its Biosynthesis-Related Genes Using Endophytic *Trichoderma harzianum* AYM3

**DOI:** 10.3390/jof9020209

**Published:** 2023-02-05

**Authors:** Adel K. Madbouly, Younes M. Rashad, Mohamed I. M. Ibrahim, Nahla T. Elazab

**Affiliations:** 1 Microbiology Department, Faculty of Science, University of Ain Shams, Cairo 4392001, Egypt; 2 Plant Protection and Biomolecular Diagnosis Department, Arid Lands Cultivation Research Institute, City of Scientific Research and Technological Applications, New Borg El-Arab 21934, Egypt; 3 National Research Centre, Food Toxicology and Contaminants Department, Giza 12622, Egypt; 4 Botany Department, Faculty of Science, Mansoura University, Mansoura 35516, Egypt

**Keywords:** mycotoxins, endophyte, biocontrol, detoxification, storage

## Abstract

Aflatoxin B1 is one of the most deleterious types of mycotoxins. The application of an endophytic fungus for biodegradation or biosuppression of AFB1 production by *Aspergillus flavus* was investigated. About 10 endophytic fungal species, isolated from healthy maize plants, were screened for their in vitro AFs-degrading activity using coumarin medium. The highest degradation potential was recorded for *Trichoderma* sp. (76.8%). This endophyte was identified using the rDNA-ITS sequence as *Trichoderma harzianum* AYM3 and assigned an accession no. of ON203053. It caused a 65% inhibition in the growth of *A. flavus* AYM2 in vitro. HPLC analysis revealed that *T. harzianum* AYM3 had a biodegradation potential against AFB1. Co-culturing of *T. harazianum* AYM3 and *A. flavus* AYM2 on maize grains led to a significant suppression (67%) in AFB1 production. GC-MS analysis identified two AFB1-suppressing compounds, acetic acid and n-propyl acetate. Investigating effect on the transcriptional expression of five AFB1 biosynthesis-related genes in *A. flavus* AYM2 revealed the downregulating effects of *T. harzianum* AYM3 metabolites on expression of *aflP* and *aflS* genes. Using HepaRG cell line, the cytotoxicity assay indicated that *T. harazianum* AYM3 metabolites were safe. Based on these results, it can be concluded that *T. harzianum* AYM3 may be used to suppress AFB1 production in maize grains.

## 1. Introduction

Maize (*Zea mays* L.) is one of the most important cereal crops worldwide, ranking first with a total global production of 1.16 billion tons [1]. It is an important oily and economic crop and has been utilized as human food and animal feed [2]. However, maize is highly susceptible to mycotoxin contaminants, including aflatoxins (AFs) [3].

Aflatoxins are potent mycotoxins produced by different mold fungi, mainly members of the genus *Aspergillus*, which cause spoilage of foods and feeds, stored commodities, and several agricultural products and lead to severe economic losses [4,5]. AFs are among the most well-known effective carcinogenic compounds, which are responsible for about 28% of the recorded cases of liver cancer worldwide [6]. Furthermore, the consumption of AF-contaminated foods causes immune-system dysfunction, acute poisoning, and stunted growth in children. *Aspergillus flavus* is the main causal agent of invasive and non-invasive aspergillosis in immunocompromised patients [7]. It can infect a variety of oilseed crops such as maize, cotton, and peanuts, leading to significant economic global losses [8]. Accordingly, *A. flavus* and AFs represent not only a real threat to human health but also cause significant economic losses in several countries as well [9].

There are different types of AFs, including AFB1, AFB2, AFG1, and AFG2. Aflatoxin B1 (AFB1) is one of the most toxic types of AFs and possesses several detrimental properties such as mutagenicity, carcinogenicity, hepatotoxicity, teratogenicity, nephrotoxicity, and immune-suppressive activities [10,11]. It is classified by the International Agency for Research on Cancer (IARC) as a class 1A carcinogen [12]. AFB1 is a major contaminant of foodstuffs and animal feeds worldwide [13]. AFs are highly stable compounds, which makes them difficult to remove from contaminated foodstuff and feed through conventional chemical and physical control methods. In addition, the complete exclusion of these mycotoxigenic fungi from the crop fields is not feasible [14,15], so a significant concern has been given to controlling the production of these AFs before harvest [16]. Numerous control methods have been employed to prevent *A. flavus* growth and subsequent AF production [8] and to remove and/or degrade these AFs in the food products [13]. These methods include the prevention of the fungal infection of crops by applying non-toxigenic strains of *A. flavus*, *A. parasiticus*, and yeasts; enhancing the host plant resistance; controlling the postharvest growth of fungi; and preventing AF production through the use of microorganisms as biocontrol agents, in addition to the use of natural products [17].

Maize is usually exposed to infection by the aflatoxigenic *A. flavus* during preharvest and postharvest stages [18]. Thus, the infection starts in the field and continues until the maize is consumed. Accordingly, it is necessary to control maize infection by *A. flavus* to prevent the accumulation of AF in the field [19]. Currently, chemical control is the main choice for control of *A. flavus*. However, chemical fungicides have many disadvantages, mainly their high cost and the fact that they are non-ecofriendly. In addition, *A. flavus* may develop a resistance to these fungicides [20]. Alternatively, the biocontrol strategy possesses an attractive and eligible alternative for the removal and degradation of AFB1 from the agricultural products due to its economic feasibility, sustainability, and safety. Furthermore, biocontrol methods maintain the safety, sensory quality, nutritional value, and acceptability of agricultural products [21].

Endophytes are defined as fungi and/or bacteria that colonize and live within plant tissues, asymptomatically for at least for a part of their life cycle, without causing any harm to the host plant [22]. They have been reported as excellent producers of novel and bioactive secondary metabolites. Secondary metabolites are small organic compounds that are not directly involved in normal microbial growth or reproduction, but they have important roles in signaling, development, and interaction with other microorganisms [23].

Several studies [24,25] have revealed that bacterial strains, including *Stenotrophomonas maltophilia* strain 35-3 and *Lactobacillus plantarum* strain FJS003, exhibited the ability to detoxify AFB1, which may be attributed to the presence of detoxifying enzyme(s) in the culture supernatant and to the strong antifungal potential against the aflatoxigenic *A. flavus* and its conidia, respectively.

*Trichoderma* spp., including *T. harzianum*, are antagonistic fungi that may naturally compete with the important phytopathogenic and mycotoxin-producing fungi and, hence, can be used as effective biocontrol agents. These fungi can produce various antifungal substances, such as harzianic acid, which act as effective antibiotics against different pathogenic fungi, such as *Pythium irregulare*, *Rhizoctonia solani*, and *Sclerotinia sclerotiorum* [26]. A recent study [27] tested 65 isolates of *Trichoderma* that belong to about 23 species for their AFB1-degrading potentials in a broth medium. *Trichoderma reesei* CGMCC3.5218 presented the best degrading capacity recording 50 ng kg^−1^ AFB1 within 3 days (100%) and 10 μg kg^−1^ AFB1 within 5 days (87.6%). In vitro, the anti-spore germination effect of *T. harzianum* HL1 and/or *T. viride* HL5 against uredospores of *Puccinia graminis* f. sp. *tritici* was reported [28]. In addition, the application of these fungi and colonization with mycorrhizal fungi significantly reduced the disease’s severity and triggered antioxidant responses in wheat against the stem rust. Inhibition of different fungal species by *T. harzianum* is comparable and more sustainable and may even be more effective than the chemical fungicides [29]. In this regard, different antagonistic modes of action have been discussed, including competition, antibiosis, mycoparasitism, and/or induction of plant defense responses [30]. Ren et al. [4] reported several *Trichoderma* spp. as potent biocontrol agents against *A. flavus*. A previous study [31] reported that pre-harvest biocontrol of *A. flavus* using potent strains of *T. harzianum* in combination with other postharvest control strategies may reduce grain contamination with aflatoxins. Several recent studies have revealed that the use of bioagents and natural products may inhibit the production of AFB1 through the downregulation of its biosynthesis genes, although the molecular mechanism of this process has not yet been understood [32].

In order to increase the shelf life of stored maize grains and replace the use of the dangerous chemical fungicides, the objectives of this study were to (1) inhibit the fungal growth of *A. flavus*; (2) suppress its AFB1 production using an endophytic fungal strain; (3) investigate the utilized mechanisms for AFB1 suppression; and (4) assess the cytotoxicity of the metabolites produced by this endophyte as a primary step for its proposed application on maize grains.

## 2. Materials and Methods

### 2.1. Collection of Maize Plants and Grains

About 20 samples of apparently healthy maize plants, including roots, stems, and leaves, were collected from maize fields in El-Sharkia Governorate, Egypt. They were placed in polyethylene bags and then transferred to the microbiology laboratory, Faculty of Science, University of Ain Shams, Cairo, Egypt. In addition, about 40 diseased grains from maize plants showing typical symptoms of *Aspergillus* ear rot were also collected and transferred to the Lab.

### 2.2. Isolation and Phenotypic Identification of the Endophytic Fungi and the Ear Rot Pathogen and Extraction of AFB1

Different endophytic fungi were isolated from the roots, stems, and leaves of the collected maize plants following the method adopted by Chatterjee [33], with slight modifications. The samples were surface-sterilized using 70% ethanol for 30 s and a 1% NaOCl for 2–3 min and washed 3 times with sterilized water. The samples were cut into small pieces (1 cm^2^) with a sterile scalpel, and then about 50 segments were aseptically placed on malt extract (ME) agar plates, supplemented with 150 μg mL^−1^ of streptomycin. After incubation for 72–96 h at 28 °C, the emerging fungal hyphae from the segments were picked up onto new plates and then purified using the single spore technique. The pure fungal cultures were kept on ME slants at 4 °C. Occurrence (%) of each fungal species was determined using the following equation:

$$\mathrm{Occurrence}\;\mathrm{of}\;a\;\mathrm{fungus}\;\left(\%\right)=\frac{\mathrm{Number}\;\mathrm{of}\;\mathrm{the}\;\mathrm{fungus}-\mathrm{infected}\;\mathrm{segments}}{\mathrm{Total}\;\mathrm{number}\;\mathrm{of}\;\mathrm{segments}\;\mathrm{examined}\;}\times 100$$

The developing fungi were identified according to their cultural, morphological, and microscopical characteristics according to Leslie and Summerell [34] and Samson et al. [35].

For isolation of the ear rot pathogen, the collected diseased maize grains were disinfected in the same manner as previously described for the healthy maize plant parts, aseptically placed on Potato dextrose agar (PDA) (Difco Laboratories, Franklin Lakes, NJ, USA) plates (pH 5.6) amended with rose bengal (0.15 g L^−1^) in triplicate, and then incubated at 28 °C for 5–7 days [36]. About 5 grains were placed on each PDA plate. After incubation, the developing fungi were purified by the single spore technique and then morphologically identified as described above. The recovered *A. flavus* AYM2 was inoculated into 250 mL of potato dextrose broth (PDB) and incubated statically at 28 °C for 5 days. After incubation, the broth culture was filtered using a muslin cloth and then centrifuged at 10,000× *g* for 10 min.

For AF extraction, 250 mL of methanol: water (55:45) was added to 50 mL of the culture filtrate, 4 g of sodium chloride, and 100 mL of n-hexan. The solution was blended for 1 min and filtered using Whatman No. 4 filter paper. About 25 mL of the filtrate was transferred to a separating funnel, 25 mL of CHCl_3_ was added, and the solution was shaken for 1 min. The CHCl_3_ layer was evaporated under N_2_ gas until dryness, and the CHCl_3_ residue was weighted. The residual sample was derivatized using 0.1 mL of trifluoroactic acid anhydride (TFA), left for 15 min at room temperature, and then 0.9 mL of acetonitrile: water (1:9) was added. AFB1 was quantified in the sample using the high-performance liquid chromatography (HPLC) according to AOAC [37].

### 2.3. HPLC Conditions

For AFs analysis using HPLC, the column used was Agilent C18 (4.6 mm × 250 mm i.d., 3.5 μm). The mobile phase was water: methanol: acetonitrile (60:30:10), and the flow rate was 1 mL min^−1^. The injection volume was 20 μL for each sample solution. The fluorescence detector was adjusted at 360/450 nm (Excitation/Emission), while the column temperature was maintained at 40 °C.

### 2.4. Screening for the AFs-Degrading Capability of the Endophytic Fungi In Vitro

The recovered endophytic fungi were screened for their capability to degrade coumarin (used as the sole carbon source), the main constituent of the AFs in vitro. Coumarin degradation was spectrophotometerically measured, according to the method of Ali et al. [38], with slight modifications. About 1 mL of a conidial suspension (2 × 10^6^ cfu mL^−1^) of each endophyte was individually inoculated into 250 mL PDB and then incubated for 5 days at 28 °C. The coumarin stock solution (1%), prepared using 10% Di-methyl sulphoxide (DMSO), was aseptically added to the broth medium to obtain final concentrations of 0.05, 0.1, 0.2, 0.3, and 0.5% and then incubated for another 72 h. After incubation, the PDB media were centrifuged at 10,000× *g* for 10 min, and the residual coumarin was extracted using chloroform. The extracted coumarin was re-dissolved in 10% DMSO, and then the absorbance was measured at 320 nm using a spectrophotometer (Robonik, India). A calibration curve was constructed using different concentrations of coumarin solution (in DMSO). PDB media supplemented with different coumarin concentrations without the endophytic conidia were used as controls. The grown isolates on coumarin at 0.5% were selected as potent isolates for AFs degradation.

### 2.5. Molecular Identification and Phylogenetic Analysis

Genomic DNAs of *A. flavus* AYM2 and the selected endophytic isolate AYM3 were extracted using the QiAamp DNA Mini Kit (Qiagen, Hilden, Germany) according to White et al. [39]. The internal transcribed spacer (ITS) region (600 bp) of the DNA was amplified using the universal primers ITS1 (5′TCCGTAGGTGAACCTTGCGG3′) and ITS4 (5′TCCTCCGCTTATTGATATGC3′). Using the NCBI search tool BLAST, nucleotide sequences of the produced amplicons were aligned and compared with the GenBank database. Phylogenetic analysis of the obtained sequences was performed using MEGA X software version 10.2.4 and compared to the closest sequences from the GenBank, and the phylogenetic tree was generated using the maximum-likelihood method [40].

### 2.6. Assessment of the Antifungal Potential of T. harzianum AYM3 against A. flavus AYM2 In Vitro

Using the dual culture technique, a 5 mm mycelial disc from a culture of *T. harzianum* AYM3 was inoculated 1 cm from the edge of a PDA plate. A 5 mm mycelial disc was cut from a 5-day-old culture of *A. flavus* AYM2 and placed 1 cm from the opposite edge of the same plate. For the control plate, a mycelial disc of *A. flavus* AYM2 was singly inoculated onto a PDA plate. The plates were incubated at 28 ± 2 °C for 7 days. The assay was carried out in triplicate and repeated twice. The inhibition percentage in mycelial growth (%) was calculated as described by Rahman et al. [41],

$$\mathrm{Growth}\;\mathrm{inhibition}\;\left(\%\right)=\frac{R1-R2}{R1}\times 100$$

where R1 is the radial growth (mm) of *A. flavus* AYM2 in the control plate and R2 is the radial growth of *A. flavus* AYM2 in the treated plate.

### 2.7. Biodegradation of AFB1 by T. harzianum AYM3

Detection of the biodegradation potential of AFB1 by *T. harzianum* AYM3 was carried out according to the method of Abdel-Shafi et al. [42], with slight modifications. The AFB1 standard solution (Sigma-Aldrich, St. Louis, MO, USA) was diluted with chloroform to a stock solution of 500 ng mL^−1^. This stock solution was aseptically added to the *T. harzianum* AYM3 PDB culture to reach a final concentration of 100 ng mL^−1^. For 96 h, the culture was statically incubated in darkness at 30 °C. As a control, sterile PDB supplemented with a diluted AFB1 standard solution was used. After 0, 12, 24, 48, and 96 h of incubation, 1 mL of the culture was centrifuged at 10,000× *g* for 10 min. AFB1 was extracted from the supernatants and quantified using HPLC, and the degradation percentage (%) was determined.

### 2.8. Effect of Co-Culturing of T. harzianum AYM3 and A. flavus AYM2 on AFB1 Production

A crushed maize grain-based medium was prepared. About 100 g of crushed grains was added to 30 mL of dist. water in 250 mL Erlenmeyer flasks and left overnight at room temperature. The flasks were steamed for 10 min without pressure and autoclaved for 30 min at 121 °C on three successive days [43]. The maize grains were then aseptically co-inoculated with two agar discs (5 mm) from the actively growing margins of *A. flavus* AYM2 and *T. harzianum* AYM3 cultures. The flasks were incubated in the dark at 30 °C for 7 days with daily shaking. Maize grains inoculated only with a single disc of *A. flavus* AYM2 served as a control. The assay was carried out in triplicate. After incubation, the AFB1 was extracted and quantified using HPLC.

### 2.9. Expression Profiling of AF Biosynthesis-Related Genes

Extraction of the total RNA from the fungal mycelium of *A. flavus* AYM2, treated and untreated with secondary metabolites of *T. harzianum* AYM3, was carried out using the RNeasy Mini Kit (Qiagen, Hilden, Germany). A reverse transcription reaction was performed for cDNA synthesis using a SureCycler 8800 (Agilent, Santa Clara, CA, USA). The reaction mixture for cDNA synthesis (20 μL) contained 3 μL RNA (30 ng), 3.8 μL RNase free water, 3 μL 5× reaction buffer, 7 μL oligo (dT) primer (5 pmol μL^−1^), 3 μL dNTPs (10 mM), and 0.2 μL reverse transcriptase enzyme (New England Biolabs, Frankfurt am Main, Germany). The reaction was processed at 42 °C for 1 h and then at 70 °C for 10 min. The product was kept at −80 °C.

A quantitative Real-Time PCR (qPCR) analysis was performed using a Rotor-Gene-6000-system (Qiagen, Valencia, CA, USA). The reaction mixture (20 μL) contained 3 μL cDNA, 12.5 μL 2XSYBR^®^ Green RT Mix (Bioloine, Brandenburg, Germany), 1.5 μL forward primer and 1.5 μL reverse primer (10 pmol μL^−1^), and 1.5 μL RNase-free water. The amplification process was programmed as follows: one cycle at 95 °C for 3 min, followed by 45 cycles (95 °C for 15 s for the denaturation step, 56 °C for 30 s for the annealing step, and 72 °C for 30 s for the elongation step). Sequences of the used primers are presented in Table 1. β-tubulin (β-tub) was used as a reference housekeeping gene. The relative expression was calculated using the comparative CT method (2^−∆∆CT^) [44]. For each sample, triplicate biological and technical replications were applied.

### 2.10. Detection of the Bioactive Anti-Aflatoxigenic Compounds from the Culture Filtrate of T. harzianum AYM3

Secondary metabolites produced by *T. harzianum* AYM3 were extracted from its culture filtrate using ethyl acetate (EA). Approximately 40 mL of EA was mixed with 100 mL of the culture filtrate and vigorously shaken for 15 min. The upper EA portion was separated using a separating funnel. The EA extract was then subjected to thin-layer chromatographic (TLC) analysis to separate its components. On the TLC plate (MERCK Silica gel F254), around 30 spots of the EA extract were made. For each spot, approximately 10 μL of the extract was used. A solution of EA and chloroform (1:1, *v*/*v*) was used as a running solvent. The developed bands were detected under UV light at 254 nm and were individually collected by scratching the gel and re-dissolving in EA. After 24 h of incubation at room temperature with mild shaking, the bioactive fractions were collected by centrifugation, followed by evaporation to dryness, according to the method adopted by Suebrasri et al. [45] with slight modifications. The dried fractions were re-dissolved in 10% DMSO and individually screened for their anti-aflatoxigenic potential. For assessment of the anti-aflatoxigenic potential, the tested fraction was aseptically added to a PDB culture of *A. flavus* AYM2 at 4:1 *v*/*v* and statically incubated in darkness at 28 ± 2 °C for 7 days. Another *A. flavus* AYM2 culture incubated without the bioactive fraction served as a control. The bioactive fractions were tested singly and/or in combination to detect any synergistic effects among them. The AFB1 was extracted and quantified using HPLC compared to the control as previously described.

### 2.11. Gas Chromatography/Mass Spectrometry (GC/MS) Analysis

The selected anti-aflatoxigenic fraction was identified using a GC/MS-QP2010 system (Shimadzu, Kyoto, Japan) equipped with a mass-selective detector (MS) and a capillary column (DB-5HT) (15 m × 0.32 mm × 0.1 µm). The detector voltage of 75 eV was used at a max temperature of 250 °C. The oven temperature was kept at 50 °C (1 min), increased to 180 °C at the rate of 15 °C min^−1^, held for 1 min, increased again to 230 °C at the rate of 7 °C min^−1^, held for 2 min, and then increased to 250 °C at 10 °C min^−1^. The injectable volume was 1.5 μL. The retention time and mass spectra of the detected components were identified by comparing them with the database of the National Institute of Standards and Technology (NIST 11) Spectral Library (Gaithersburg, MD, USA).

### 2.12. Cytotoxicity of the Secondary Metabolites of T. harzianum AYM3

The cytotoxicity of the secondary metabolites of *T. harzianum* AYM3 was tested against the human hepatocyte (HepaRG) cell line using the methyl-thiazolyl-tetrazolium (MTT) assay according to Roopan et al. [46]. The HepaRG cell line was kindly provided by the VACSERA Institute, Dokki, Giza, Egypt. The cell line was prepared by dissolving it in 0.1% DMSO. A mixture of Dulbecco’s Modified Eagle Medium (DMEM) supplemented with 10% of heat-inactivated fetal bovine serum (FBS), 2 mM l-glutamine, 100 IU mL^−1^ of penicillin, and 100 mg mL^−1^ of streptomycin antibiotics was used for growing the HepaRG cells. The cells were maintained in a 5% CO_2_ humidified incubator at 37 °C. The growing cells were harvested and then seeded in 96-well plates (Thermo Scientific, Braunschweig, Germany) at 1 × 10^5^ cells/well. After 24 h of incubation, the HepaRG cells were cleaned twice with 100 μL of serum and then starved at 37 °C for 1 h. After starvation, the HepaRG cells were incubated in different concentrations of the *T. harzianum* AYM3 culture filtrate, specifically 10, 25, 50, 100, 200, and 400 μg mL^−1^ at 37 °C for 72 h. The serum-free medium containing 0.5 mg mL^−1^ of MTT was added after aspiration and incubated at 37 °C for 4 h in a CO_2_-humidified incubator. Three replicate wells were used for each concentration. Doxorubicin was used as the positive control treatment. Finally, the cells were washed using 200 μL of phosphate-buffered saline. The developing crystals were dissolved by adding 100 μL of 0.1% DMSO, and the optical density was determined for each well at 570 nm using an ELX 808 Ultra Microplate Reader (Biotek, Winooski, VT, USA). Cell viability (%) was determined compared with the untreated cells. The half-maximal inhibitory concentration (IC_50_) values were calculated for the metabolites of *T. harzianum* AYM3 and the control doxorubicin.

### 2.13. Statistical Analyses

Data were analyzed using the software CoStat (version 6.4, https://www.cohortsoftware.com/costat.html, accessed on 12 May 2022). Comparisons between means were performed using Tukey’s HSD test at *p* ≤ 0.05 based on one-way ANOVA.

## 3. Results

### 3.1. Isolation and Identification of the Endophytic Fungi and the Ear Rot Pathogen

Isolation of the endophytic fungi from the roots, stems, and leaves of maize plants led to the recovery of 10 fungal isolates belonging to eight different genera. The isolated endophytic fungi were morphologically identified as *Fusarium graminearum* (18%), *Fusarium* sp. (12%), *A. niger* (23%), *Macrophomina* sp. (9%), *A. fumigatus* (12%), *Trichoderma* sp. (14%), *Penicillium* sp. (10%), *Cheatomium* sp. (7%), *Curvularia* sp. (11%), and *Mucor* sp. (5%). Moreover, an isolate of *A. flavus* was recovered from the diseased maize grains, which was designated as AYM2.

**Figure 1:**
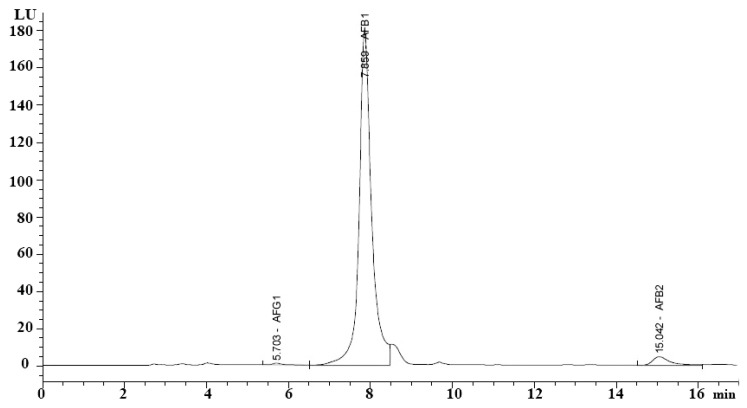
HPLC chromatogram showing AFs produced by *A. flavus* AYM2. AFB_1_, AFB_2_, and AFG_1_ refer to aflatoxins B_1_, B_2_, and G_1_, respectively. LU: refers to the luminescence unit.

Results obtained from the HPLC analysis (Figure 1) indicated the ability of *A. flavus* AYM2 to produce three types of AFs at varying levels, namely AFB1 (141.52 ng mL^−1^), AFB2 (4.38 ng mL^−1^), and AFG1 (0.98 ng mL^−1^).

### 3.2. Screening for the AFs-Degrading Capability of the Endophytic Fungi In Vitro

**Figure 2:**
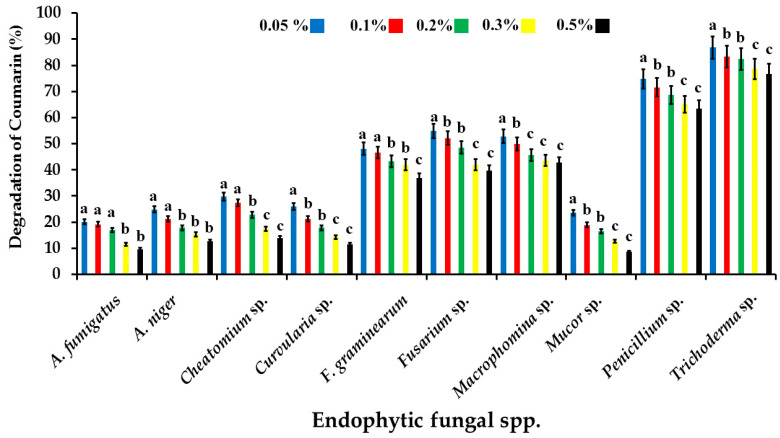
Coumarin-degradation activity of different endophytic fungi at 0.05, 0.1, 0.2, 0.3, and 0.5% coumarin on coumarin agar medium. The error bars represent standard errors. Columns superscripted with the same letter are not significantly different according to Tukey’s HSD test at *p* ≤ 0.05. Each value represents the mean of three replicates.

All 10 fungal isolates were able to degrade the coumarin at all tested concentrations, at varying extents. However, the degradation activity decreased with the increment in the coumarin concentration for all tested fungi. The highest degrading activity at 0.5% coumarin was recorded for *Trichoderma* sp. (76.8%), followed by *Penicillium* sp. (62.4%), while that of *Fusarium* sp. and *Macrophomina* sp. was recorded at 54.8 and 52.8%, respectively. *Curvularia* sp. and *Mucor* sp. showed the lowest degrading activity, recording 11.6 and 8.6%, respectively (Figure 2). Based on these results, most of the recovered endophytic fungi were excluded due to their probable pathogenicity (i.e., *Macrophomina* sp. and *Curvularia* sp.), and/or mycotoxin-producing capabilities (i.e., *F. graminis*, *Fusarium* sp., *Penicillium* sp., and *A. niger*). However, *Trichoderma* sp. was selected for further study, as it exhibited effective biodegradation of coumarin (the main component of AFB1) in vitro and is known to be non-aflatoxigenic. This promising isolate was designated as AYM3.

### 3.3. Molecular Identification and Phylogenetic Analysis

**Figure 3:**
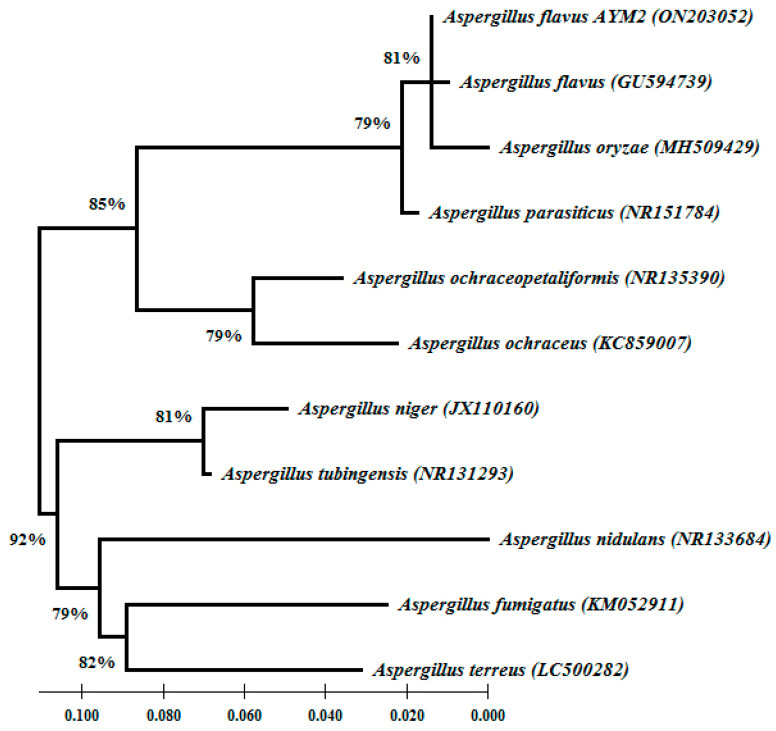
Phylogenetic tree showing the relationship between *Aspergillus flavus* AYM2 and ten different species of Aspergillus genus based on the GenBank database (accession numbers are shown). The phylogenetic tree was generated using the maximum-likelihood method [34] with 1000 bootstrap replicates. Bootstrap values ≥ 79% are illustrated on nodes. The scale bar represents the number of nucleotide substitutions per site.

**Figure 4:**
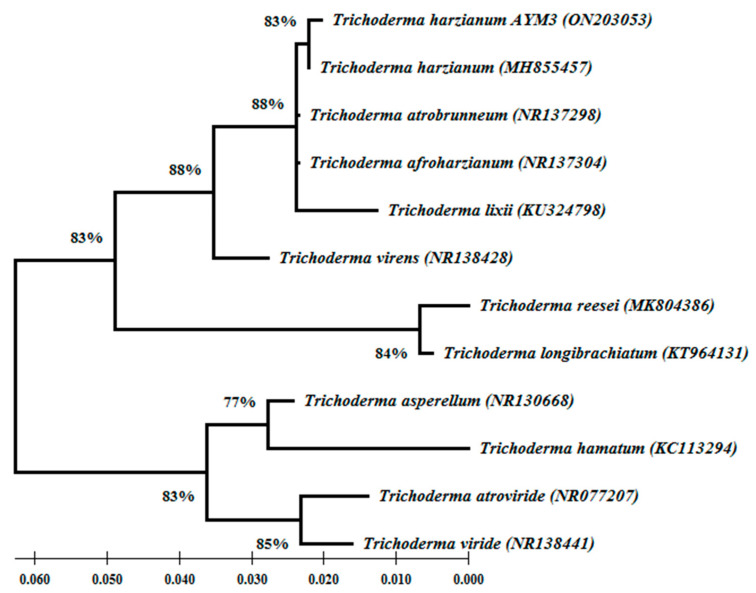
Phylogenetic tree showing the relationship between *Trichoderma harzianum* AYM3 and eleven different species of Trichoderma genus based on the GenBank database (accession numbers are shown). The phylogenetic tree was generated using the maximum likelihood method [34] with 1000 bootstrap replicates. Bootstrap values ≥ 77% are illustrated on nodes. The scale bar represents the number of nucleotide substitutions per site.

Based on the rDNA-ITS sequences, BLAST analysis results indicate that the sequence of the tested fungus AYM2, isolated from infected maize grains, showed a high similarity (100%) with *A. flavus* (MT645322). It also showed that the sequence of the endophytic fungus AYM3, isolated from maize roots, stems and leaves, exhibited a high similarity (100%) with *T. harzianum* (MH855457). The nucleotide sequences of *A. flavus* AYM2 (531 bp) and *T. harzianum* AYM3 (549 bp) were deposited in the GenBank under accession numbers ON203052 and ON203053, respectively. Phylogenetic analysis of the ten different species of the *Aspergillus* genus demonstrated that all species were clustered into two main groups. The first group contained two clades; one of them contained *A. flavus* AYM2, *A. flavus,* and *A. oryzae* in one subclade with 81% bootstrap support, while the other subclade contained *A. parasiticus*. In the second clade, *A. ochraceus* and *A. ochraceopetaliformis* were clustered with each other with 79% bootstrap support. In the second main group, two clades were clustered with 92% bootstrap support. In one clade, *A. fumigatus*, *A. terreus*, and *A. nidulans* were grouped, while the second clade contained *A. niger* and *A. tubingensis* with 81% bootstrap support (Figure 3). Regarding *T. harzianum* AYM3, phylogenetic analysis of eleven different species of the Trichoderma genus revealed that it was grouped with *T. harzianum* in one distinct clade with 83% bootstrap support. The species were clustered into two main groups. The first group contained *T. harzianum*, *T. afroharzianum*, *T. atrobrunneum*, and *T. lixii* in one clade with 88% bootstrap support. In the other clade, *T. reesei* and *T. longibrachiatum* were clustered with 84% bootstrap support. While *T. virens* was used as an outgroup, in the second main group, *T. asperellum* and *T. hamatum* were clustered in a separate clade with 77% bootstrap support, and the other clade contained *T. viride* and *T. atroviride* with 85% bootstrap support (Figure 4).

### 3.4. Antifungal Activity of T. harzianum AYM3 against A. flavus AYM2

Endophytic *T. harzianum* AYM3 was tested for its antagonistic activity against *A. flavus* AYM2 in vitro. Results obtained from the dual culture assay showed that *T. harzianum* AYM3 was faster in its growth rate than *A. flavus* AYM2. After 5 days of incubation, a clear zone was formed between the two colonies limiting the mycelial growth of *A. flavus* AYM2. On day 7, a significant inhibition (65%) in the growth of *A. flavus* AYM2 was recorded, compared to the control. After ten days of incubation, *A. flavus* AYM2 was overgrown by *T. harzianum* AYM3. The obtained results revealed the antagonistic activity of *T. harzianum* AYM3 against *A. flavus* AYM2.

### 3.5. Biodegradation of AFB1 by T. harzianum AYM3

**Figure 5:**
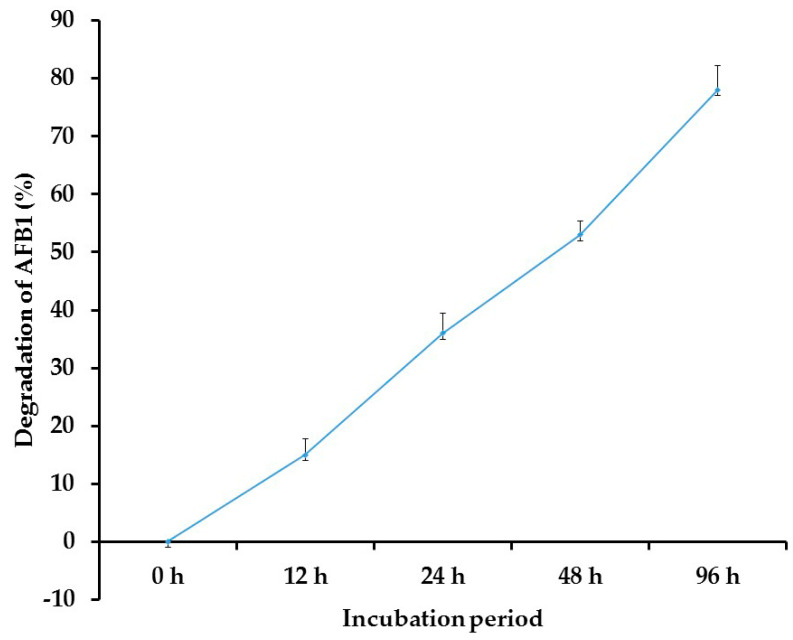
Degradation of AFB1 by *Trichoderma harzianum* AYM3 within 96 h of incubation. Each value represents the mean of three replicates. The error bars represent the standard errors, where *p* ≤ 0.05.

Data from the HPLC analysis revealed that *T. harzianum* AYM3 had a biodegrading potential against AFB1 in vitro. Moreover, the degrading activity increased with the increment in the incubation period (Figure 5). The highest activity was observed after 96 h of incubation, recording 78% degradation, where *p* ≤ 0.05. In contrast, the heat-treated culture did not record any degradation level.

### 3.6. Effect of Co-Culturing of T. harzianum AYM3 and A. flavus AYM2 on AFB1 Production

**Figure 6:**
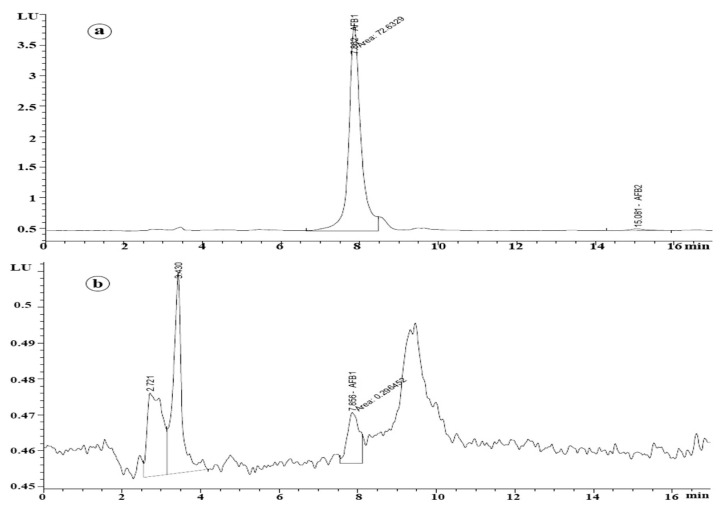
HPLC chromatogram showing AFB_1_ produced by *A. flavus* AYM2 on maize grains (**a**) and reduced AFB_1_ level in response to co-culturing of *T. harzianum* AYM3 and *A. flavus* AYM2 (**b**).

The effect of co-culturing of *T. harzianum* AYM3 and *A. flavus* AYM2 on AFB1 production on maize grains was assessed. Results obtained from the HPLC analysis showed a considerable reduction (67%) in the AFB1 level, compared to the control treatment (Figure 6a,b).

### 3.7. Expression Profiling of AF Biosynthesis-Related Genes

**Figure 7:**
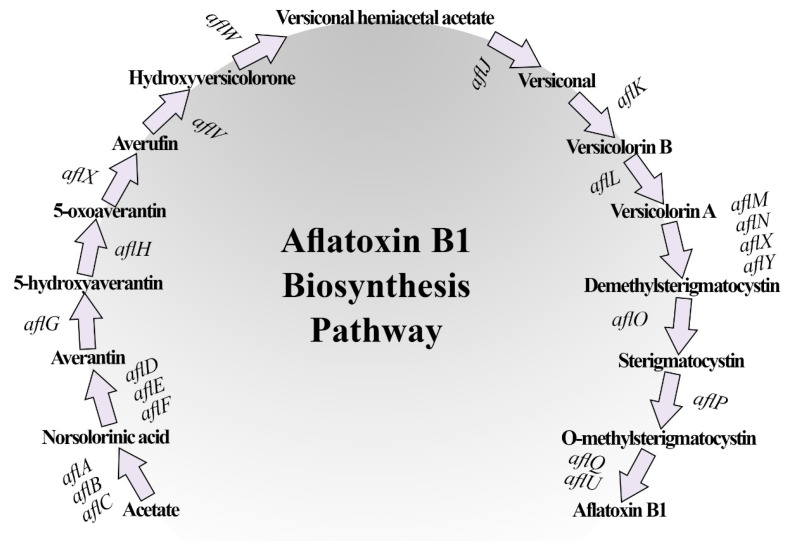
A schematic diagram of the biosynthesis pathway of aflatoxin B1, indicating the corresponding encoding genes. Modified from [47].

**Figure 8:**
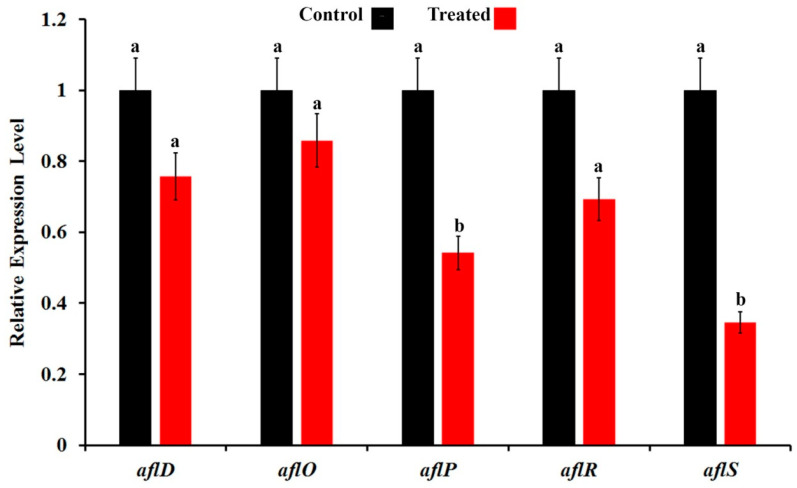
A histogram showing expression profiles of 5 AF biosynthesis-related genes and transcriptional factors in *A. flavus* AYM2 (*aflD*, *aflO*, *aflP*, *aflR*, and *aflS*) in response to treatment with secondary metabolites produced by *T. harzianum* AYM3. For each gene, columns superscripted with the same letter are not significantly different according to Tukey’s HSD test at *p* ≤ 0.05. Values are means of 3 biological replicates, and each sample was analyzed in triplicate. Error bars represent standard errors.

Aflatoxin B1 biosynthesis pathway stages with their corresponding encoding genes are illustrated in Figure 7. The effect of secondary metabolites produced by *T. harzianum* AYM3 on transcriptional expression level of 5 aflatoxin biosynthesis-related genes and transcriptional factors in *A. flavus* AYM2, namely *aflD*, *aflO*, *aflP*, *aflR*, and *aflS*, is illustrated in Figure 8. The obtained results indicated that treating *A. flavus* AYM2 with the culture filtrate of *T. harzianum* AYM3 significantly suppressed the expression of *aflP* and *aflS*, while the reported downregulation in gene expression of *aflD*, *aflO*, and *aflR* was not significant. The expressions of *aflD* (0.758-fold), *aflO* (0.858-fold), *aflP* (0.542-fold), *aflR* (0.692-fold), and *aflS* (0.346-fold) were also obtained.

### 3.8. TLC Analysis of T. harzianum AYM3 Metabolites

TLC analysis separated the metabolites produced by *T. harzianum* AYM3 into six fractions. All separated fractions were tested for their anti-aflatoxigenic potential against *A. flavus* AYM2. Results obtained showed that two fractions (1 and 2) only suppressed AFB1 production by *A. flavus* AYM2, recording a 61 and 33% reduction, respectively. Meanwhile, the combination of both bioactive compounds at a ratio of 1:1 and incubation with *A. flavus* AYM2 PDB culture exerted a synergistic potential, as they reduced the AFB1 level by 70%. Both fractions were mixed and subjected to GC-MS analysis to identify their composition, while the remaining fractions exhibited weak anti-aflatoxigenic potential.

### 3.9. GC-MS Analysis

**Figure 9:**
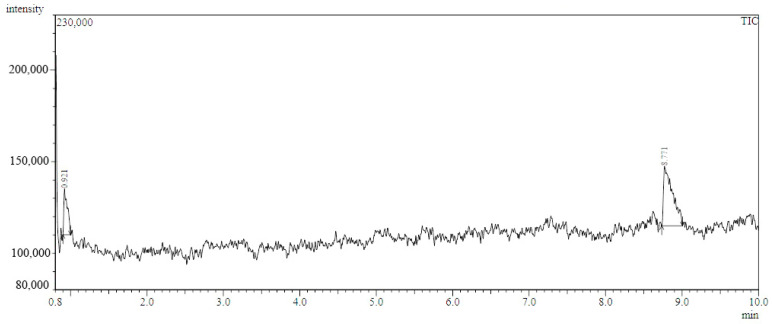
GC-MS chromatogram showing the chemical composition of the mixed bioactive fractions separated from the culture filtrate of *T. harzianum* AYM3.

Data obtained from GC-MS analysis showed that the mixed fractions contained two bioactive compounds (Figure 9). Compound (A) was identified as acetic acid, the major component (76.2%) at a retention time of 8.771, and compound (B) was identified as n-propyl acetate (23.8%) at a retention time of 0.921, the minor component.

### 3.10. Cytotoxicity of Secondary Metabolites of T. harzianum AYM3

**Figure 10:**
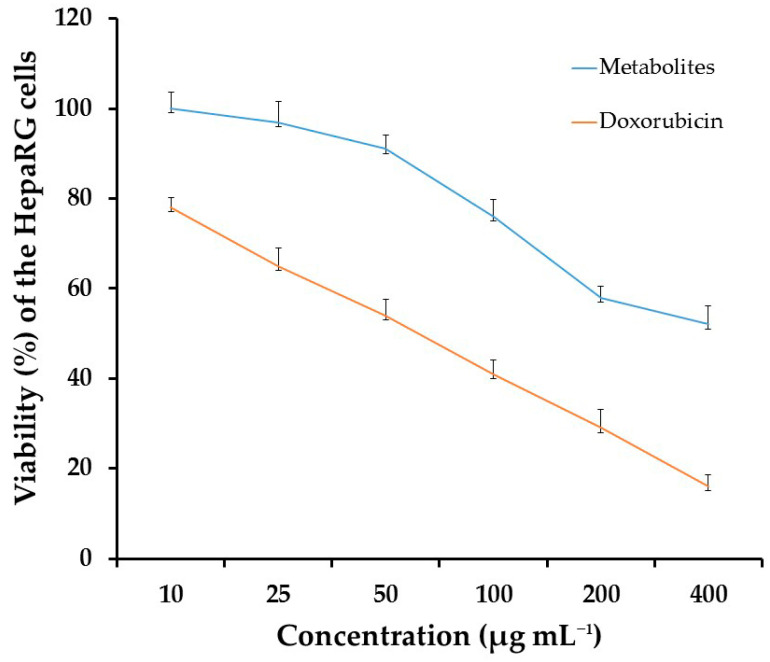
Cytotoxicity of secondary metabolites produced by *T. harzianum* AYM3 compared to doxorubicin at different concentrations. The error bars represent the standard errors, where *p* ≤ 0.05.

Cytotoxicity of the secondary metabolites produced by *T. harzianum* AYM3 on the HepaRG cells is illustrated in Figure 10. The metabolites of *T. harzianum* AYM3 showed a concentration-dependent cytotoxic effect. Results indicated that metabolites of *T. harzianum* AYM3 had no toxicity at 10 μg mL^−1^. However, viability of the HepaRG cells decreased with the increment in concentration of metabolites of *T. harzianum* AYM3, reaching the minimum point at 400 μg mL^−1^, recording 52% viability, while doxorubicin recorded 78% viability at 10 μg mL^−1^ and reached the most toxic point at 400 μg mL^−1^, recording 16% viability, where *p* ≤ 0.05. The recorded IC_50_ values of *T. harzianum* AYM3 metabolites and doxorubicin for HepaRG cells were 397 and 48 µg mL^−1^, respectively.

## 4. Discussion

AFB1 is one of the most toxic types of Afs; it contaminates foodstuffs and animal feeds and causes mutagenic, carcinogenic, teratogenic, hepatotoxic, and immune-suppressive effects [10]. In this study, the application of endophytic fungi for biodegradation and/or biosuppression of AFB1 production by *A. flavus*, the main AFB1 producer on maize grains, was investigated. In this regard, a total of 10 endophytic fungal species belonging to eight genera were isolated. The highest prevalence was recorded for *A. niger* (23%), followed by *F. graminearum* (18%), and *Trichoderma* sp. (14%). This result is in agreement with that obtained by Potshangbam et al. [48], who reported members of the genera *Fusarium*, *Aspergillus*, and *Penicillium* as the most commonly isolated fungal endophytes from maize plants. HPLC analysis from this study showed a high level of AFB1 production by *A. flavus* AYM2, isolated from the rotted maize grains. Various studies have reported *A. flavus* and *A. parasiticus* as the highest AFs producers among the aflatoxigenic fungi at a wide range of temperatures and water activity [49].

Although all of the tested endophytic fungi showed varying levels of coumarin degrading potential, *T. harzianum* AYM3 outperformed them all. This result is in accordance with the result obtained by Hackbart et al. [50], who reported a 100% degradation of AFB1 in a PDA plate by *T. reesii* QM9414 after 4 days of incubation. However, microbial degradation using different types of microorganisms has been extensively studied. Because the AFB1 molecule is a highly substituted coumarin derivative (difuranocoumarin) with a coumarin ring and a lactone moiety in its structure, a coumarin medium has been used to screen microorganisms for AFB1-degrading ability [25]. A previous study conducted by Guan et al. [24] reported that microorganisms that can metabolize coumarin as a sole carbon source may be able also to metabolize aflatoxins, mainly AFB1. In this study, the recorded coumarin-degrading activity of *T. harzianum* AYM3 revealed that the AFB1 could be effectively detoxified by this strain, which can be attributed to the action of one or more of its produced enzymes that catalyze the coumarin-ring cleavage. The enzymatic mechanism of AFB1 biodegradation by *T. harzianum* AYM3 was confirmed later, as the heat-treated culture filtrate of this strain did not record any biodegrading potential. In this regard, multiple enzymes have been reported to exhibit AF degrading activity, such as aflatoxin oxidase [51], laccases [52], and manganese peroxidases [53]. However, a microbial non-enzymatic mechanism was also reported. Yao et al. [54] attributed the AFB1 transformation activity of *Pseudomonas* strain m29 to its metabolites 1,2-dimethylhydrazine or 1,1-dimethylhydrazine.

Using the dual culture technique, *T. harzianum* AYM3 exhibited a significant antifungal potential against *A. flavus* AYM2, recording 65% growth inhibition. The strong antifungal potential expressed by *T. harzianum* AYM3 against *A. flavus* AYM2 growth demonstrated that AFB1 production could be significantly inhibited by this strain, in accordance with findings of Zhu et al. [25]. Furthermore, this result is also in agreement with the recent study conducted by Metz and Hausladen [55], where six *Trichoderma* strains inhibited the growth of three *Alternaria solani* strains by 35–85% within 5 days of confrontation. Several previous studies recorded the competition exerted by *Trichoderma* spp. in vitro, which inhibited the fungal growth of *Macrophomena phaseolina* and *R. solani* [56], *Corynespora cassiicola* and *C. aeria* [57], and *Stagonosporopsis cucurbitacearum* [58]. This inhibitory effect can be attributed to different modes of action, including competition for nutrients and/or space, which was supported in the present study by the fast growth rate of *T. harzianum* AYM3, compared to that of *A. flavus* AYM2. Moreover, antibiosis can be another possible mechanism of *T. harzianum* AYM3 via the production of inhibitory volatile, non-volatile compounds, and/or antifungal enzymes. This mechanism was supported by the observed inhibition zone limiting the growth of *A. flavus* AYM2. In addition, mycoparasitism may contribute to the antagonistic behavior of *T. harzianum* AYM3 towards *A. flavus* AYM2, as it showed an overgrowth on *A. flavus* AYM2 after 10 days of incubation of the dual culture.

One of the most interesting results obtained in this study is the degrading effect of *T. harzianum* AYM3 on AFB1, recording a 78% reduction after 96 h. This result is in agreement with that obtained in several previous studies. Adebo et al. [59] found that although AFs are produced by several fungi, certain species, including *A. parasiticus*, *A. flavus*, *Absidia repens*, *Candida utilis*, *Paecilomyces lilacinus*, *Dactylium dendroides*, *Penicillium* spp., *Mucor* spp., *Peniophora* spp., *Pleurotus ostreatus*, *Phoma* spp., *Rhizopus* spp., and *Trichoderma* spp. have the ability to degrade these AFs. In this study, AFB1 biodegradation may be attributed to the production of AF-degrading enzymes, including coumarinases, laccases, peroxidases, and oxidases. Various bioactive metabolites from fungi, bacteria, and algae have been widely reported to degrade AFs [59]. In this regard, El-Shiekh et al. [60] reported a 67.2% inhibition in AFs production of *A. parasiticus* RCMB 002001(2) by co-culturing with *T. viride*. The degradation of the cyclopentane ring in the AF molecule, forming a furan moiety, was detected, indicating the enzymatic degradation mechanism.

Results obtained from this study revealed that co-culturing of *T. harazianum* AYM3 and *A. flavus* AYM2 on maize grains led to a significant suppression (67%) in AFB1 production. This suppressive activity can be attributed to the secondary metabolites produced by *T. harazianum* AYM3, which may inhibit AFB1 synthesis via the downregulation of the AFB1-biosynthesis-related genes or may detoxify (degrade) the produced AFB1. Different microbial bioactive metabolites have been reported as AFB1 synthesis inhibitors, such as organic acids, which reduce the medium pH to an extent sufficient to suppress AFB1 biosynthesis. Xing et al. [9] recorded a downregulation of AFB1 biosynthesis-related genes by *A. flavus* when co-cultured with *A. niger*. In addition, the degradation of produced AFB1 was also reported and attributed to lactonase and reductase activity of *A. niger* metabolites. Furthermore, a recent study conducted by Iannaccone et al. [61] reported that *Trichoderma* spp. are capable of synthesizing several enzymes—mainly chitinase, β-1, 3-glucanase, phenylalanine ammonia-lyase, and peroxidase—to withstand the adverse environmental conditions. As an interpretation of the results recorded in the current study, a previous work [50] revealed the involvement of peroxidases in AFB1 and ochratoxin degradation, as these enzymes have the power to oxidize the AFs into less toxic and polar derivatives [62]. The background peaks observed in Figure 6b represent the derivatives obtained on biodegradation of AFB1 by *T. harzianum* AYM3 when co-cultured with *A. flavus* AYM2 on maize grains.

The effect of secondary metabolites produced by *T. harzianum* AYM3 on the transcriptional expression level of five AF biosynthesis-related genes and transcriptional factors (*aflD*, *aflO*, *aflP*, *aflR*, and *aflS*) in *A. flavus* AYM2 was investigated. The obtained results from the qPCR showed that treating *A. flavus* AYM2 with the culture filtrate of *T. harzianum* AYM3 significantly suppressed the expression of *aflP* and *aflS* genes to varying extents, indicating its anti-aflatoxigenic activity. In a recent study, Ren et al. [4] investigated the antagonistic activity and inhibitory metabolites of 20 *Trichoderma* isolates against growth and AFB1 production by the aflatoxigenic *A. flavus* ITEM 9. Among them, two anti-aflatoxigenic isolates were selected and tested for the suppression of the AF biosynthesis-related genes as a probable mechanism for the inhibition of AF production. Although both of the selected *Trichoderma* isolates significantly reduced AFB1 production, neither isolate could suppress the AF synthesis genes. They concluded that the reduction in AFB1 content might be due to the enzymatic degradation or complexation. In our study, we reported the suppression effect on AFB1 synthesis by secondary metabolites of *T. harzianum* AYM3 via the downregulation of some genes in the AF biosynthesis cluster as a mechanism of its anti-aflatoxigenic potential. To the best of our knowledge, this is the first report of suppression of AF synthesis genes by *Trichoderma* sp. as an anti-aflatoxigenic mechanism. *aflD* is a structural gene in the early stages of the AF biosynthesis pathway encoding a keto reductase enzyme, which catalyzes the conversion of norsolorinic acid to averantin. This gene is considered a marker for differentiation between aflatoxigenic and non-aflatoxigenic isolates [63]. Regarding *aflO*, it is a structural gene in the intermediate stages of the AF biosynthesis pathway and encodes for O-methyl transferase (I) enzyme, which catalyzes the conversion of dimethyl-sterigmatocystin into sterigmatocystin [64]. Furthermore, *aflP* is another structural gene that encodes for an O-methyl transferase (II) enzyme, which is involved in the conversion of sterigmatocystin into O-methylsterigmatocystin [65]. *aflR* it is a regulatory gene (zinc finger transcription factor) that mainly regulates more than 17 genes in the AF biosynthetic cluster, which encode an enzymatic cascade resulting in the biosynthesis of different AFs [66]. Meanwhile, *aflS* is a putative transcription factor that is located next to *aflR* in the cluster and acts as a co-activator for *aflR* [67]. The proposed functional mechanism includes the formation of a protein dimer between *aflR*’s and *aflS*’s corresponding proteins, which enhances the successful binding to the gene promoter of the AF biosynthesis-related genes [68]. The currently recognized reduction in AFB1 level may be attributed to the downregulation of *aflS*, which leads to a reduction in the ratio of *aflS*: *aflR* transcripts and hence the protein–dimer ratio, which negatively affected gene expression in the AF biosynthesis cluster, resulting in a reduction in AF production. In this regard, Chang et al. [69] reported a downregulation of up to 20-fold in the transcriptional level of the AF biosynthesis-related genes *aflC*, *aflD*, *aflM*, and *aflP* in *aflS* knockout mutants of *A. parasiticus*. Furthermore, a downregulation in expression of *aflS* and *aflP* genes, which is reported in this study, may explain the observed reduction in AF production in *A. flavus* AYM2.

GC-MS analysis demonstrated that the two anti-aflatoxigenic metabolites detected in the bioactive fractions of *T. harzianum* AYM3 were identified as acetic acid (76.2%) and n-propyl acetate (23.8%). This result is consistent with that obtained by Moon et al. [70], who reported a complete inhibition of AFB1 production via the downregulation of aflatoxin biosynthesis-related genes in *A. flavus* ATCC 22546 as a response to treatment with 0.5% acetic acid. In accordance with the results of this study, a significant suppression in the transcriptional expression level of *aflE*, *aflL*, *aflO*, *aflQ*, *aflR*, and *aflS* genes was observed in *A. flavus* ATCC 22546 treated with acetic acid at 0.05 and 0.1%. Moreover, detoxification of AFs using organic acids such as acetic and lactic acids has been reported. In spite of the high thermal stability of AFB1, it has been shown that acetic acid can partially degrade AFB1 at 80 °C [71].

Results from this study demonstrated that the metabolites of *T. harzianum* AYM3 had no to low cytotoxic activity on HepaRG cells, especially at low concentrations, compared to the doxorubicin that was used as a positive control treatment. On increasing the concentrations of this CFS, the HepaRG cells’ viability decreased, presumably due to the effect of the secondary metabolites such as acetic acid present in the CFS on the cell components. However, this decrease in viability was low. Conversely, upon increasing the concentrations of the doxorubicin, the HepaRG cells’ viabilities decreased significantly, indicating the lethal effect of this doxorubicin control treatment on the HepaRG cells. Moreover, at high concentrations of 200 and 400 μg L^−1^, the cytotoxic effect of *T. harzianum* AYM3 metabolites was almost half of that recorded for the doxorubicin treatment. Furthermore, the IC_50_ values for *T. harzianum* AYM3 metabolites and control doxorubicin were 397 ± 2 and 48 ± 2 μg mL^−1^, respectively, indicating the low toxicity effects of the metabolites on the HepaRG cells. Accordingly, the metabolites of *T. harzianum* AYM3 may be used effectively and safely to inhibit the growth of *A. flavus* AYM2 and to suppress the AFB1 production in stored maize grains instead of the deleterious chemical fungicides.

## 5. Conclusions

To the best of our knowledge, this study reported for the first time the suppressive effect of *T. harzianum* AYM3 metabolites (i.e., acetic acid and n-propyl acetate) on the transcriptional expression of AFs biosynthesis-related genes and transcriptional factors. Moreover, both metabolites expressed synergistic activity. These metabolites synergistically downregulated the expression of *flP* and *aflS* genes. In addition, the detoxifying (degrading) effect of *T. harzianum* AYM3 metabolites on AFB1 was also reported. An antagonistic effect was also observed against the mycelial growth of *A. flavus* AYM2 in vitro. Furthermore, using the human HepaRG cell line, the results of the cytotoxicity test revealed that *T. harzianum* AYM3 metabolites are safe, at least at low concentrations. Based on the obtained results, *T. harzianum* AYM3 strain represents a promising bioagent, as it exhibited several inhibitory modes of action against *A. flavus* AYM2 growth, suppression of production, and biodegradation of AFB_1_. Accordingly, this potent strain may be used to suppress AFB1 production, as well as detoxify this mycotoxin in maize grains during storage, thus acting as an effective alternative to synthetic fungicides.

## Figures and Tables

**Table 1:** Primer sequences of the genes of interests.

Gene Code	Accession No.	Sequence (5′–3′)
*aflD*-F	AFLA_139390	AGGCATCTGTGCTCGGATTG
*aflD*-R		TGCCCCGATGTAGTCTCCTTAGT
*aflO*-F	AFLA_139220	CCCCAAGAGTATACCTCGAGTGC
*aflO*-R		AAGGTCCCGAGATGTCGAATAGTT
*aflP*-F	AFLA_139210	TATTCTACATGACTATCCCGATGCTG
*aflP*-R		GCGCGACTTGCTTGGGT
*aflR*-F	AFLA_139360	GCGGCACAGCTTGTTCTGA
*aflR*-R		CCGGTATCCCTGCTGCATC
*aflS*-F	AFLA_139340	AACGGTCGTGCATGTGGG
*aflS*-R		CGGCCTTAGCTTCTGTCTGC
*β-tub*-F	AFLA_068620	AACGTCTACTTCAACGAGGCCA
*β-tub*-R		GTACCAGGCTCAAGATCAACGAG

## Data Availability

All data generated or analyzed during this study are included in this published article.
